# Vascular Calcification Progression Modulates the Risk Associated with Vascular Calcification Burden in Incident to Dialysis Patients

**DOI:** 10.3390/cells10051091

**Published:** 2021-05-03

**Authors:** Antonio Bellasi, Luca Di Lullo, Domenico Russo, Roberto Ciarcia, Michele Magnocavallo, Carlo Lavalle, Carlo Ratti, Mario Cozzolino, Biagio Raffaele Di Iorio

**Affiliations:** 1 Department of Medicine, Division of Nephrology, Ente Ospedaliero Cantonale, 6900 Lugano, Switzerland; 2 Department of Nephrology and Dialysis, Ospedale Parodi, Delfino, Colleferro, 00034 Rome, Italy; luca.dilullo@aslroma5.it; 3 Department of Nephrology, School of Medicine, University of Naples Federico II, 80137 Naples, Italy; domenico.russo2@unina.it; 4 Departments of Veterinary Medicine and Animal Productions, University of Naples Federico II, 80137 Naples, Italy; roberto.ciarcia@unina.it; 5 Department of Clinical, Internal, Anesthesiology and Cardiovascular Sciences, Policlinico Universitario Umberto I, Sapienza University of Rome, 00161 Roma, Italy; michele.magnocavallo@uniroma1.it (M.M.); carlo.lavalle@uniroma1.it (C.L.); 6 Department of Cardiology, Ospedale Ramazzini Carpi, 41012 Carpi, Italy; c.ratti@ausl.mo.it; 7 Renal Division, Department of Health Sciences, ASST Santi Paolo e Carlo, University of Milan, 20122 Milan, Italy; mario.cozzolino@unimi.it; 8 Nefrology and Dialysis, AORN “San Giuseppe Moscati”, 83100 Avellino, Italy; br.diiorio@gmail.com

**Keywords:** coronary artery calcification, progression, hemodialysis, risk prediction

## Abstract

*Background*: It is estimated that chronic kidney disease (CKD) accounts globally for 5 to 10 million deaths annually, mainly due to cardiovascular (CV) diseases. Traditional as well as non-traditional CV risk factors such as vascular calcification are believed to drive this disproportionate risk burden. We aimed to investigate the association of coronary artery calcification (CAC) progression with all-cause mortality in patients new to hemodialysis (HD). *Methods*: Post hoc analysis of the Independent study (NCT00710788). At study inception and after 12 months of follow-up, 414 patients underwent computed tomography imaging for quantification of CAC via the Agatston methods. The square root method was used to assess CAC progression (CACP), and survival analyses were used to test its association with mortality. *Results*: Over a median follow-up of 36 months, 106 patients died from all causes. Expired patients were older, more likely to be diabetic or to have experienced an atherosclerotic CV event, and exhibited a significantly greater CAC burden (*p* = 0.002). Survival analyses confirmed an independent association of CAC burden (hazard ratio: 1.29; 95% confidence interval: 1.17–1.44) and CACP (HR: 5.16; 2.61–10.21) with all-cause mortality. CACP mitigated the risk associated with CAC burden (*p* = 0.002), and adjustment for calcium-free phosphate binder attenuated the strength of the link between CACP and mortality. *Conclusions*: CAC burden and CACP predict mortality in incident to dialysis patients. However, CACP reduced the risk associated with baseline CAC, and calcium-free phosphate binders attenuated the association of CACP and outcomes, suggesting that CACP modulation may improve survival in this population. Future endeavors are needed to confirm whether drugs or kidney transplantation may attenuate CACP and improve survival in HD patients.

## Study Highlights

- Coronary artery calcification (CAC) burden portends poor prognosis (hazard ratio (HR) for all-cause mortality: 1.29; 95% confidence interval (95% CI): 1.17–1.44) in incident to dialysis patients.
- CAC progression (CACP) portends poor prognosis (HR for all-cause mortality: 5.16; 2.61–10.21) in incident to dialysis patients.
- CACP mitigated the risk associated with CAC burden (*p* = 0.002).
- Calcium-free phosphate binder is associated with survival benefit.
- Adjustment for calcium-free phosphate binder attenuated the strength of the link between CACP and mortality, suggesting that CACP modulation may improve survival in incident to dialysis patients.

## 1. Introduction

Chronic kidney disease (CKD) is believed to affect 8% to 15% of the population and it is estimated to account globally for 5 to 10 million deaths annually [1,2,3,4]. In these regards, cardiovascular disease (CVD) is the most common cause of death [4,5]. Though the mechanisms largely await elucidation, traditional as well as non-traditional cardiovascular (CV) risk factors are believed to drive this disproportionate CVD burden [6,7].

In CKD patients, the occurrence of vascular calcification (VC) is two- to five-fold more common than in age-matched subjects with preserved renal function [8,9]. Several methods are available to quantitate VC in different arterial sites and have been used to address the prognostic value of VC in various low- to high-risk populations [9]. A convincing body of evidence supports the notion that coronary artery calcification (CAC) detected by means of cardiac computed tomography (CCT) correlates with atherosclerotic plaque burden and risk of CV events in both non-dialysis-dependent and dialysis-dependent CKD subjects [8,9]. Indeed, the findings from the Chronic Renal Insufficiency Cohort (CRIC) study suggested that CAC is strongly associated with the occurrence of myocardial infarction, heart failure and stroke in CKD Stages 2–4 [10,11]. Although limited by the small sample, a seminal work by Block and coworkers documented an independent and graded association of CAC burden and risk of all-cause mortality in a cohort of patients starting hemodialysis [12]. Of interest, the reported association of VC with outcome was independent of adjustment for various confounding factors, supporting its use for risk prognostication in CKD subjects [11].

Whether the serial CAC assessment adds to a single evaluation of CAC burden is a matter of an intense debate. In a large cohort of 4609 asymptomatic individuals from the general population, CAC progression added incremental value in predicting all-cause mortality over the baseline score and several other confounders [13]. We sought to evaluate the factors associated with CAC progression as well as the independent association of CAC progression, baseline CAC and all-cause mortality in a large cohort of 414 subjects new to hemodialysis (less than 3 months).

## 2. Material and Methods

### 2.1. Study Cohort and Endpoint of Interest

We utilized data from incident hemodialysis patients recruited in the Independent study (*ClinicalTrials.gov: NCT00710788*). A detailed description of the study protocol and cohort has already been provided [14,15]. Briefly, the independent study was designed to assess the impact of 2 different phosphate binder regimens (calcium-free vs. calcium-containing phosphate binder) on CV events as well as all-cause mortality. As per the study protocol, sevelamer HCl as a calcium-free phosphate binder, and calcium carbonate or calcium acetate as calcium-containing phosphate binder were administered during study follow-up. A total of 466 adult (>18 years) patients starting hemodialysis (requiring dialysis for less than 120 days) were randomized (1:1 ratio) to receive open-label sevelamer or calcium carbonate or calcium acetate as a phosphate binder regimen. The study was conducted at 18 dialysis centers in Italy. The inclusion criteria were: (i) age younger than 75 years and (ii) hemodialysis dependency for less than 120 days. Exclusion criteria were: (i) a history of cardiac arrhythmia, (ii) the syndrome of congenital prolongation of the QT segment interval, (iii) a corrected QT (QTc) longer than 440 ms or increased QT dispersion (QTd), (iv) a history of coronary artery bypass (CABG), (v) liver dysfunction and hypothyroidism, or (vii) use of drugs that prolong the QT interval [14,15]. Written informed consent was obtained from all participants prior to study entry and after approval from each institutional Ethical Review Board. The study was conducted in adherence with the Declaration of Helsinki, Ethical Principles for Medical Research Involving Human Subjects. As summarized elsewhere [15], The independent trial showed significant CV and all-cause survival benefits associated with calcium-free phosphate binders. In this post hoc analysis, we wanted to test whether CAC progression was independently associated with risk of death.

Enrollment in the independent study began in September 2006 and continued through to July 2008, and the study follow-up ended in July 2011.

During follow-up, physicians were instructed to manage patients according to the guidelines available at the time the study was conceived. In particular, these suggested controlling blood pressure (target: <130/80 mm Hg), anemia (Hb: 11 g/dL, TSAT: 20%), acidosis (HCO_3_ between 20 and 24 mmol/L), diabetes (HbA1c: <7.0%), dyslipidemia (total cholesterol: <200 mg/dL; LDL cholesterol: <100 mg/dL; triglycerides: <180 mg/dL), and the parameters of bone mineral metabolism (serum phosphorous: 2.5–5.0 mg/dL; serum calcium: 8.0–9.9 mg/dL; intact PTH between 150 and 300 pg/mL).

The endpoint of interest was defined as all-cause mortality and, by study design, all patients were followed until death or study completion (36 months of follow-up). No data regarding kidney transplantation in patients recruited in the independent study was recorded.

### 2.2. Demographic, Clinical and Laboratory Characteristics, and Vascular Calcification Assessment

Demographic, clinical and laboratory characteristics were collected at study inception. A history of atherosclerotic disease (ASCVD) was defined if any of the following clinical data were reported: a history of cerebrovascular disease, peripheral vascular disease, angina pectoris, a history of myocardial infarction, aortic aneurysm or a history of percutaneous coronary angioplasty, with or without stenting.

Routine biochemical laboratory measurements were obtained at baseline and at 6-monthly intervals. For the current analysis, only data on the baseline measurements were considered. All blood samples were taken before the midweek dialysis session and after 12 h of fasting. Serum parameters of anemia, electrolytes, mineral metabolism and dialysis adequacy were performed by the usual laboratories of the facilities.

Vascular calcification and arterial stiffness were evaluated at study entry and at 6-monthly intervals for the first 24 months after study inception. Coronary artery calcification (CAC) was assessed by multi-slice lightspeed (GE Medical Systems) equipment at one center (Solofra, Italy). A standard imaging protocol was used to acquire a set of ECG-gated tomographic slices from the carina to the diaphragm. CAC was calculated as described by Agatston et al [16] (Agatston score), computing each lesion with a radiological density of >130 HU identified along the course of the coronary arteries.

CAC progression was defined using the Agatston CAC score and according to the square root method described by Hokanson and coworkers: progression was defined if the square root of the CAC score at 12 month minus the square root of the baseline CAC score was > 2.5 (CAC score at 12 months > CAC score at baseline)] [17], because this is the most reliable tool to assess progression and it provides prognostic information [13].

Arterial stiffness was assessed through carotid–femoral pulse wave carotid femoral velocity (PWV) measurement. PWV was evaluated by applanation tonometry with a Pulse Pen (Diatecne, Milan, Italy) as previously described, namely as the ratio between the distance (in m) and the travelling time (in s) of the pulse generated in each cardiac cycle [18]. Hence, PWV is expressed as m/sand higher values represent stiffer arteries.

### 2.3. Statistical Analysis

In this post hoc analysis, no adjustment for multiple comparisons was made. Data are expressed as means (standard deviation -SD) or medians (interquartile range (IQR) as appropriate. Categorical variables are presented as proportions. Parametric (*t*-test) and non-parametric tests (Wilcoxon sum rank test, Chi square test, Fisher’s exact test) were used as appropriate to compare demographic and clinical characteristics according to the occurrence of any lethal event (status) before study completion (36 month follow-up). Owing to the skewness of the CAC score, the baseline CAC was log-transformed (log(CAC + 1)) if used as a continuous variable, or categorized as CAC = 0, CAC between 1–99, CAC between 100–399 or CAC = 400+ if used as a categorical variable [12,19].

Logistic regression models were used to detect the predictors of CAC progression. Variables forced into the fully adjusted model were selected on the basis of existing evidence. A stepwise approach was utilized to select the most parsimonious model.

Univariable- and multivariable-adjusted survival analyses were performed. By study design, all patients were followed until the occurrence of a lethal event or completion of 36 months of follow-up. The cumulative incidence of the study endpoint (all causes of death) by coronary artery calcification (CAC) strata was constructed by the Kaplan–Meier method, and the log rank test was used to determine statistical significance. Cox proportional hazard regression analyses were applied to estimate the risk of death (hazard ratios (HR)) according to baseline CAC, CAC progression and the interaction term (baseline CAC × CAC progression). Cox proportional hazard regression analyses are presented as: (1) unadjusted; (2) adjusted for age (Model 1); (3) adjusted for Model 1 and diabetes, ASCVD and systolic blood pressure (Model 2); (4) adjusted for Model 2 and pulse wave velocity and LVMI (Model 3); (5) adjusted for Model 3 and calcium-free phosphate binders (Model 4). All covariates were selected a priori as potential confounders. To better gauge the modification effect of CAC progression on baseline CAC, a similar approach was repeated for patients with and without evidence of CAC progression.

Statistical significance was set at 0.05. All analyses were completed using R version 3.6.2 (2019-12-12; R Foundation for Statistical Computing, Vienna, Austria). The following packages were used: survival, splines, epiDisplay, foreign, ggplot2, survimer and dplyr.

## 3. Results

Table 1 shows the baseline characteristics of the study participants. Data from 414 middle-aged (65.3 (14.8)) men (48.8%) and women were utilized for this analysis (88% of the original independent study cohort) (Table 1). A total of 52 patients from the original cohort were excluded due to various reasons: expiration, loss to follow-up or missing a CAC evaluation after 12 months from study initiation. Diabetes, ASCVD and baseline CAC burden were the most important differences between included and excluded patients (Supplemental Table S1).

The main characteristics of the study cohort according to status at study completion are summarized in Table 1. In the overall study cohort, ASCVD (32.6%), diabetes mellitus (28.3%) and coronary artery calcification (68.2% of the study cohort had detectable CAC) were the most common comorbid conditions (Table 1). Over a median follow-up of 36 months (interquartile range: 28–36 months), 106 patients died (all causes). Expired patients were older (71 vs. 63 years, *p* < 0.001), more likely to be diabetic (55% vs. 18%, *p* < 0.001) or to have experienced an atherosclerotic cardiovascular event (48% vs. 27%, *p* < 0.001) or exhibited a significantly greater CAC burden (*p* = 0.002) (Supplemental Figure S1). Differences in laboratory characteristics as well as use of drugs were also apparent (Table 1).

At the end of the first 12 months of follow-up, about one-third of the study cohort (33.1%) experienced significant CAC progression. Notably, the proportion of subjects experiencing CAC progression by study completion was almost two-fold greater among expired vs. survived (50.9% vs. 26.9%, *p* < 0.0001) patients, suggesting that CAC progression is associated with all-cause mortality (Table 1).

**Figure 1:**
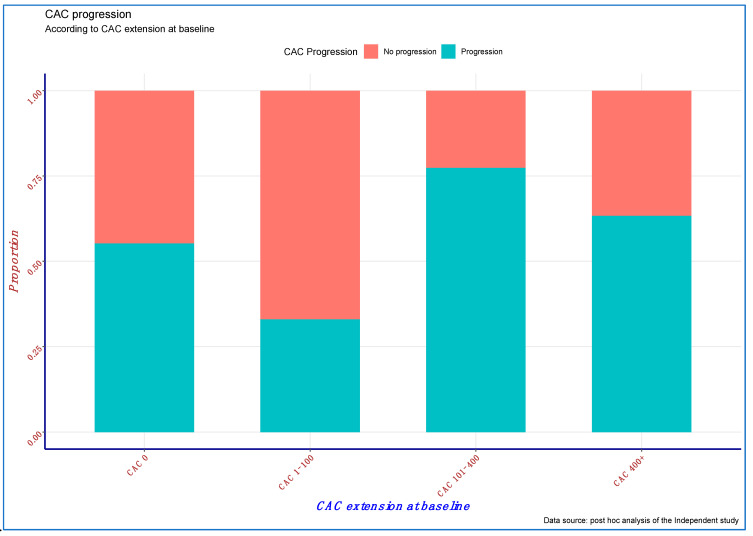
Coronary artery calcification (CAC) progression according to baseline CAC stratification.

CAC progression was directly associated with age (odds ratio (OR): 1.019, 95% confidence interval (95% CI): 1.004–1.036; *p* = 0.015) and inversely associated with the use of calcium-free phosphate binder (OR: 0.132; 95% CI: 0.063–0.279; *p* < 0.001) (Table 2). Although relevant, baseline CAC (Figure 1) was only marginally and inversely associated with the risk of CAC progression (OR: 0.921; 95% CI: 0.827–1.025; *p* = 0.131), likely due to a significant positive interaction between baseline CAC and use of calcium-free phosphate binders (OR: 1.36; 95% CI: 1.115–1.659; *p* = 0.002), which suggests a greater effect of these compounds on CAC progression prevention with a progressively higher CAC burden (Table 2).

To investigate the association between baseline CAC, CAC progression and all-cause survival, an unadjusted Cox model was first fitted and progressively adjusted for factors either associated with vascular calcification or risk of death in HD patients (Table 3). Both the baseline extent of CAC (hazard ratio (HR) per log increase in Agatston score: 1.28; 95% CI: 1.15–1.43; *p* < 0.001) and CAC progression (HR: 4.24; 95% CI: 2.16–8.33; *p* < 0.001) during the first 12 months of follow-up were significantly associated with the risk of all-cause mortality. Notably, a significant effect interaction was present (HR: 0.82; 95% CI: 0.71–0.95; *p* = 0.008), suggesting that CAC progression may attenuate the risk associated with baseline CAC. Progressive adjustment of the model for various factors (Table 3—Models 1 through 3) did not significantly affect these associations. However, CAC progression and the interaction term were significantly attenuated after adjustment for the use of calcium-free phosphate binders (Table 3—Model 4). Because these drugs are associated with both CAC progression (Table 2) and survival (Supplemental Tables S2 and S3), these results suggest that the survival benefit associated with sevelamer is partially mediated by its effect on CAC progression.

**Figure 2:**
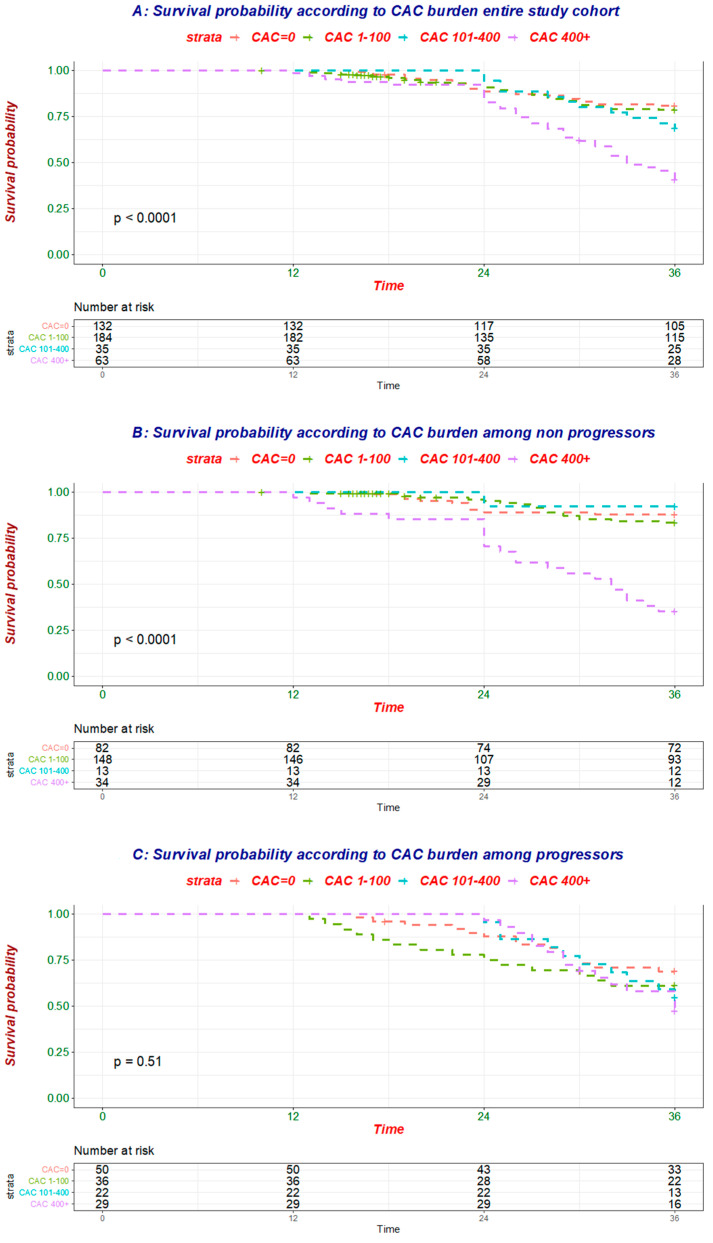
Survival probability according to baseline coronary artery calcification (CAC) in the entire study cohort (**A**) as well as patients without (**B**) and with (**C**) evidence of CAC progression during the first 12 months of follow-up.

To better understand how CAC progression influenced the relationship of baseline CAC and mortality (Figure 2A, Table 4), the association between baseline CAC and all-cause mortality was tested separately in individuals without (*n* = 277, 52 deaths; Figure 2B, Table 4) and with (*n* = 137, 54 deaths; Figure 2C, Table 4) evidence of CAC progression.

While baseline CAC was linked to mortality in the former group, no association was evident in the latter one (Table 5 and Table 6), further corroborating the finding that CAC progression modulates the risk associated with baseline CAC.

## 4. Discussion

A disproportionate cardiovascular (CV) risk in CKD has been repeatedly reported [4,5]. Of note, while in the general population, atherosclerotic events are the major driver of CV morbidity, in CKD patients, non-atherosclerotic CV events such as cardiac arrythmia and heart failure are more prevalent [4]. The generalized cardiovasculopathy described in subjects with CKD receiving dialysis reflects a complex interplay of traditional (including hypertension, dyslipidemia, diabetes mellitus, left ventricular hypertrophy, physical inactivity) as well as non-traditional (including endothelial dysfunction, inflammation, oxidative distress, uremic toxins, metabolic derangements such as mineral metabolism abnormalities, etc.) CV risk factors [6,8] Although it is unlikely that a single factor is responsible for the increased risk, a recently defined syndrome that encompasses laboratory (including serum levels of calcium, phosphate and parathyroid hormone), bone (including uremic osteodystrophy) and vascular (including vascular calcification) abnormalities called chronic kidney disease mineral metabolism disorder (CKD-MBD) may contribute to the CV fragility noted in CKD [20].

In CKD patients, the occurrence of vascular calcification (VC) is two- to five-fold more common than in age-matched subjects with preserved renal function [8,9]. As in the general population [21], a convincing body of evidence supports the notion that VC is a marker of vascular injury and portends poor survival [10,11,12]. Several tools and methods are available to quantitate VC, but cardiac computed tomography (CCT) is the actual gold standard for evaluating the extent of coronary artery calcification (CAC). Data from the Multi-Ethnic Study of Atherosclerosis (MESA) [22] demonstrated a significantly increased risk of a CV event (defined as the composite of coronary heart disease, stroke, heart failure and peripheral artery disease) per 1 standard deviation increase in the log of CAC (hazard ratio (HR): 1.69; 95% confidence interval (95% CI): 1.45–1.97) among 1284 non-dialysis-dependent CKD (NDD-CKD) subjects [22]. Notably, this association was independent of adjustments with Framingham predictors and was stronger than what reported for other markers of vasculopathy such as carotid intima–media thickness or ankle–brachial index [22]. In another cohort of 1541 participants without cardiovascular disease and CKD Stages 2–4, Chen and coworkers [10] reported that the hazard ratios per 1 SD log of CAC were 1.40 (95% CI, 1.16–1.69) for cardiovascular disease, 1.44 (95% CI, 1.02–2.02) for myocardial infarction, 1.39 (95% CI, 1.10–1.76) for heart failure and 1.19 (95% CI, 0.94–1.51) for all-cause mortality irrespective of adjustments for traditional and non-traditional CV risk factors [6]. While the data are convincing in NDD-CKD patients, evidence in dialysis patients is scanty and have been largely derived from small study cohorts. In these regards, a seminal work from Block and coworkers [12] documented a graded increase in the risk of all-cause mortality according to CAC burden in 127 subjects new to dialysis (less than 3 months). Indeed, the baseline CAC score was a significant predictor of mortality after adjustment for age, race, gender and diabetes, with increased mortality proportional to the baseline score (*p* = 0.002) [12]. In another cohort of 166 patients on chronic maintenance hemodialysis, higher baseline CAC was associated with an increased adjusted risk of death independent of demographics, comorbidity, lipids and other cardiovascular risks, surrogates of bone disease, nutritional and inflammatory markers, and dialysis dose [19].

Whether CAC progression provides additional prognostic information is still a matter of debate. In a large cohort of 4609 consecutive asymptomatic individuals without CKD referred by primary physicians for CAC measurement, serial assessment of CAC by different methods added incremental value in predicting mortality (HR individuals with vs. without CAC progression: 3.34; 95% CI: 2.65 to 4.21) [13]. In a smaller series of 181 NDD-CKD patients, rapid progression of CAC (defined as CAC progression greater than the 75th percentile of the distribution) was significantly and independently associated with the risk of myocardial infarction (HR: 6.3; 95% CI: 1.5–26.2) [23]. Of interest, in this study, both baseline CAC (HR associated with CAC > 100 AU: 8.4; 95% CI: 2.3–30.1) and CAC progression were independent predictors of CV events [23]. Furthermore, a significant interaction effect was also reported, suggesting that CAC progression reduces the risk burden associated with baseline CAC (HR: 0.08; 95% CI: 0.01–0.5) [23].

The current findings expand the available evidence, suggesting that VC progression 12 months after the first CT scan predicts all-cause survival in incident hemodialysis patients. Similar to what has been reported in NDD-CKD, CAC progression modulates and reduces the risk associated with baseline CAC extent. Indeed, stratification of the study cohort according to evidence of CAC progression yielded different results, with baseline CAC being linked with mortality only among patients with a stable CAC burden at follow-up.

Differences in plaque composition between subjects with and without renal function impairment have been reported [24,25]. While plaque density is inversely related to mortality in non-CKD patients [24], it is directly related to mortality in hemodialysis patients [25]. Accelerated vascular senescence [26] as well as CKD-MBD may explain these findings. A large body of evidence suggests that administration of calcium as a phosphate binder to control hyperphosphatemia increases the risk of VC progression [27]. Excessive calcium loading may result in hydroxyapatite crystal formation and deposition in soft tissues and arterial walls [28], further promoting VC. In our analyses, the addition of phosphate binder type (calcium-free vs. calcium-containing phosphate binders) to the survival model significantly attenuated the association of CAC progression and all-cause mortality, which lost significance. In consideration of the strong association of phosphate binder type with mortality, as well as with CAC progression, it is tempting to speculate that part of the survival benefit associated with the choice of phosphate binder is mediated by attenuation of CAC progression. Whether this is due to the additive effect of sevelamer HCl on lipids, inflammation, uric acid and fetuin-A [29], or, alternatively, to a reduction in calcium’s skeletal buffering capacity associated with CKD-MBD [28] cannot be investigated with the current data. Nevertheless, calcium intake from supplements increased the risk of CAC development and progression in subjects recruited in the MESA study [30], suggesting a role of CKD-MBD management in modulating the risk of CAC and mortality. Although tempting, whether attenuation of VC progression confers a survival benefit in patients receiving dialysis needs to be verified in future through properly designed studies. In this regard, other agents have shown promise for reducing VC progression and may be considered for future studies. In the ADVANCE study, cinacalcet, a calcium-sensing receptor (CaR) agonist, showed a trend toward reduction of CAC progression when compared with Vitamin D [31]. Matrix Gla-protein (MGP) activation by Vitamin K supplementation or the use of direct anticoagulants also show promise, and these approaches are currently under investigation [8,32]. Administration of magnesium to patients with NDD-CKD slowed CAC progression in an open-label study [33]. Finally, SNF472 (myoinositol hexaphosphate) resulted in significant slowing of CAC progression (11%: 95% CI: 7–15% vs. 20%; 95% CI 14–26%) [34,35] Moreover, future endeavors should investigate whether CAC, and to what extent CACP, is influenced by kidney transplantation. Indeed, correction of CKD-MBD or other metabolic abnormalities after renal function restoration may positively impact CACP and contribute to explaining the survival benefit associated with transplantation. However, evidence of CACP after transplantation is still controversial and far from being conclusive, since other factors such as immunosuppressants and pre-existing cardiovascular conditions may promote vascular calcification progression, even after transplantation [36].

This study suffers from a few limitations. This is a post hoc analysis of a randomized clinical trial designed to assess the impact of calcium-free vs. calcium-containing phosphate binders on CV events and survival, and unmeasured residual confounders such as use of statins or Vitamin K antagonists cannot be excluded. Nonetheless, the relatively large study cohort and the adjustments for various factors associated with either CAC or mortality suggest the robustness of these findings. The relatively small number of CV events (67 fatalities) prevented us from investigating the association of CAC or CAC progression with cardiovascular outcome. However, the use of a less accurate endpoint such as all-cause mortality should dilute rather than strengthen the associations reported. We studied a cohort of incident hemodialysis patients, and these results may not be generalized to prevalent hemodialysis or peritoneal dialysis. Nevertheless, the current results are in line with the available evidence, and the fact that demographic and comorbid conditions did not explain our findings corroborates the notion that CAC progression predicts outcomes in dialysis patients. Finally, the investigators were instructed to adjust the medications and assess the transplant eligibility of enrolled patients according to clinical guidelines available at the time the independent study was conducted. No intervention was protocolized. While this may be perceived as a study protocol limitation, the relatively small number of patients not completing the study (less than 5% per year) and the good balance of lost-to-follow-up patients across study sites suggest that the current results are unlikely to have been influenced by patients who did not complete the independent study follow-up.

In summary, we document a strong and independent association between CAC progression and mortality in a cohort of incident dialysis patients. Of interest, CAC progression modulated the risk associated with baseline CAC, which predicted the outcome only if no CAC progression occurred. Although speculative, these results also suggest that the survival benefit associated with calcium-free vs. calcium-containing phosphate binders is partly explained by their effect on CAC progression. Future studies are required to confirm whether drugs or kidney transplantations which correct metabolic abnormalities in CKD also attenuate vascular calcification progression and improve survival in patients starting dialysis.

## Figures and Tables

**Table 1:** Patients’ characteristics according to study cohort and status at study completion.

Variable	Total (*n* = 414)	Alive (*n* = 308)	Expired (*n* = 106)	
	Mean (SD)(n)	Mean (SD)(*n*)	Mean (SD)(*n*)	*p*-Value
Age (years)	65.3 (14.8)(414)	63.1 (14.8)(308)	71.5 (12.9)(106)	<0.0001
Male (%)	48.8% (202)	46.4% (143)	55.7% (59)	0.127
Body Weight (kg)	70.7(13.7)(414)	72.8 (13.3)(308)	64.7 (13.0)(106)	<0.0001
ASCVD (%)	32.6% (135)	27.3% (84)	48.1% (51)	<0.0001
Diabetes (%)	28.3% (117)	18.8% (58)	55.7% (59)	<0.0001
Systolic Blood Pressure (mmHg)	137 (18)(414)	136 (17)(308)	140 (19)(106)	0.056
Diastolic Blood Pressure (mmHg)	76 (9)(414)	76 (8)(308)	76 (10)(106)	0.741
LVMI (g/cm^2^)	149 (45)(414)	146 (48)(308)	158 (34)(106)	0.007
QTc (ms)	407 (32)(414)	406 (34)(308)	410 (26)(106)	0.314
QTd (ms)	26 (11)(414)	27 (11)(308)	25 (11)(106)	0.193
CAC Agatston score (unit)	273 (728)(414)	181 (633)(308)	542 (903)(106)	0.0002
CAC strata				<0.0001
CAC = 0	31.8 (132)	34.7 (107)	23.5 (25)	
CAC 1–99	44.4 (184)	49.0 (151)	31.1 (33)	
CAC 100–399	8.4 (35)	7.7 (24)	10.3 (11)	
CAC 400+	15.3 (63)	8.4 (26)	34.9 (37)	
CAC Agatston score progression	33.1% (137)	26.9% (83)	50.9% (54)	<0.0001
Pulse Wave Velocity (m/s)	8.7 (2.4)(414)	8.5 (1.8)(308)	9.4 (3.7)(106)	0.012
Hemoglobin (g/dL)	11.0 (1.4)(414)	11.1(1.4) (308)	10.9(1.5)(106)	0.269
Albumin (g/dL)	3.8 (0.4)(414)	3.9 (0.4)(308)	3.6 (0.4)(106)	<0.0001
Total Cholesterol (mg/dL)	161(48)(414)	158(44)(308)	165(53)(106)	0.192
LDL Cholesterol (mg/dL)	100(29)(414)	98(28)(308)	102(31)(106)	0.168
Triglycerides (mg/dL)	175(111)(414)	179(126)(308)	170(89)(106)	0.402
Serum Creatinine (g/dL)	7.9 (2.6)(414)	8.0 (2.5)(308)	7.4 (2.5)(106)	0.019
Hemoglobin (g/dL)	11 (1.4)(414)	10.9 (1.4)(308)	11.2 (1.4)(106)	0.045
Sodium (mE/L)	139 (3.2)(413)	138 (3.4)(307)	139 (2.7)(106)	0.0005
Potassium (mEq/L)	5.2 (0.8)(414)	5.2 (0.8)(308)	5.1 (0.7)(106)	0.117
Calcium (mg/dL)	8.9 (0.7)(413)	8.9 (0.8)(307)	8.6 (0.6)(106)	<0.0001
Phosphate (mg/dL)	5.2 (1.5)(413)	5.3 (1.6)(307)	4.9 (1.4)(106)	0.015
Parathyroid Hormone (pg/mL)	271 (207)(414)	277 (204)(308)	255 (215)(106)	0.363
C-reactive protein (mg/L)	7.7 (11.2)(414)	8.4 (12.4)(308)	5.5 (6.5)(106)	0.003
Use of ACE Inhibitors (%)	79.5% (329)	79.9% (246)	78.3% (83)	0.837
Use of ARBs (%)	88.2% (365)	86% (265)	94.3% (100)	0.035
Use of Beta Blockers (%)	50.2% (208)	54.2% (167)	38.7% (41)	0.008
Use of Calcium Channel Blockers (%)	32.4% (134)	29.2% (90)	41.5% (44)	0.027
Use of Cinacalcet (%)	52.4% (217)	52.9% (163)	50.9% (54)	0.811
Use of Vitamin D (%)	43.2% (179)	43.2% (133)	43.4% (46)	1
Use of Sevelamer (%)	50.7% (210)	42.2% (130)	75.5% (80)	<0.0001
Use of Calcium-Based Binders (%)	49.3% (204)	57.8% (178)	24.5% (26)	<0.0001

ASCVD: atherosclerotic cardiovascular disease was defined if any of the following clinical data were reported: a history of cerebrovascular disease, peripheral vascular disease, angina pectoris, a history of myocardial infarction, aortic aneurysm or a history of percutaneous coronary angioplasty with or without stenting. CAC: coronary artery calcification; ACE: angiotensin converting enzyme; ARB: angiotensin receptor blocker; LDL: low density lipoprotein cholesterol.

**Table 2:** Predictors of coronary artery calcification (CAC) progression by logistic regression analysis. (A) Fully adjusted model; (B) most parsimonious model selected according to a stepwise approach.

		95% Confidence Interval		
	Odds Ratio	Lower Boundary	Upper Boundary	
Baseline coronary artery calcification [log(CACstart + 1)]	0.913	0.817	1.021	0.110
Use of calcium-free phosphate binder (yes vs. no)	0.128	0.059	0.279	<0.001
Pulse wave velocity at baseline (m/s)	0.988	0.9	1.085	0.796
Age (years)	1.022	1.006	1.039	0.008
History of diabetes (yes vs. no)	0.691	0.411	1.162	0.163
History of ASCVD (yes vs. no)	1.096	0.679	1.767	0.708
Interaction term (baseline CAC × calcium-free phosphate binder)	1.381	1.125	1.70E + 00	0.002
		**95% Confidence Interval**		
	**Odds Ratio**	**Lower Boundary**	**Upper Boundary**	
Baseline coronary artery calcification [log(CACstart + 1)]	0.921	0.827	1.025	0.131
Use of calcium-free phosphate binder (yes vs. no)	0.132	0.063	0.279	<0.001
Age (years)	1.019	1.004	1.036	0.015
Interaction term (baseline CAC × calcium-free phosphate binder)	1.36	1.115	1.659	<0.001

ASCVD, atherosclerotic cardiovascular disease, defined if any of the following clinical data were reported: a history of cerebrovascular disease, peripheral vascular disease, angina pectoris, a history of myocardial infarction, aortic aneurysm or a history of percutaneous coronary angioplasty, with or without stenting.

**Table 3:** Predictors of all-cause mortality in the overall study cohort.

Predictors of All-Cause Mortality (Cox Model)—All Subjects *n* = 414 (106 Fatalities)				
		95% Confidence Interval		
Model	HR	Lower Boundary	Upper Boundary	Pr(>|z|)
**Unadjusted**				
Baseline CAC score (log CAC +1) per log increase	1.32252	1.1855	1.4754	<0.001
CAC progression (yes vs. no)	4.2082	2.1258	8.3307	<0.001
Interaction term	0.7978	0.6913	0.9207	0.002
**Model 1: adjusted for age**				
Baseline CAC score (log CAC +1) per log increase	1.3024	1.1671	1.4533	2.34 × 10^−6^
CAC progression (yes vs. no)	4.1393	2.093	8.1863	4.45 × 10^−5^
Interaction term	0.7939	0.6881	0.9159	0.00156
**Model 2: adjusted for Model 1 + diabetes + ASCVD + systolic blood pressure**				
Baseline CAC score (log CAC +1) per log increase	1.2876	1.1565	1.4335	3.96 × 10^−6^
CAC progression (yes vs. no)	4.2444	2.1608	8.3371	2.71 × 10^−5^
Interaction term	0.8268	0.7172	0.9531	0.00876
**Model 3: adjusted for Model 2 + PWV + LVMI**				
Baseline CAC score (log CAC +1) per log increase	1.2987	1.171	1.4402	7.34 × 10^−7^
CAC progression (yes vs. no)	5.165	2.6128	10.2101	2.33 × 10^−6^
Interaction term	0.8019	0.6965	0.9232	0.00213
**Model 4 adjusted for Model 3 + use of calcium-free phosphate binder**				
Baseline CAC score (log CAC +1) per log increase	1.1287	1.0114	1.2596	0.03055
CAC progression (yes vs. no)	1.9591	0.9214	4.1652	0.08058
Interaction term	0.96	0.8255	1.1164	0.59595

ASCVD, atherosclerotic cardiovascular disease, defined if any of the following clinical data were reported: a history of cerebrovascular disease, peripheral vascular disease, angina pectoris, a history of myocardial infarction, aortic aneurysm or a history of percutaneous coronary angioplasty with or without stenting. PWV: pulse wave velocity; LVMI: left ventricular mass index; HR: hazard ratio.

**Table 4:** Survival probability according to baseline coronary artery calcification (CAC) in the entire study cohort (A) as well as patients without (B) and with (C) evidence of CAC progression during the first 12 months of follow-up.

Entire Study Cohort			Confidence Interval		
	Survival (%)	St Err	Lower 95%	Upper 95%	
CAC = 0	0.86	0.03	0.85	0.92	
CAC 1–100	0.84	0.02	0.8	0.913	
CAC 101–400	0.87	0.05	0.77	0.99	
CAC 400+	0.56	0.06	0.44	0.712	
No Evidence of CAC Progression					
CAC = 0	0.94	0.02	0.9	0.99	
CAC 1–100	0.89	0.02	0.83	0.95	
CAC 101–400	0.92	0.07	0.78	1	
CAC 400+	0.44	0.09	0.29	0.67	
Evidence of CAC Progression					
CAC = 0	0.72	0.06	0.6	0.86	
CAC 1–100	0.71	0.07	0.57	0.88	
CAC 101–400	0.83	0.08	0.67	1	
CAC 400+	0.69	0.09	0.53	0.89	

**Table 5:** Predictors of all-cause mortality among individuals without evidence of coronary artery calcification (CAC) progression.

Predictors of All-Cause Mortality (Cox Model)—Non-Progressor Subjects *n* = 277 (52 Fatalities)				
Model	HR	95% Confidence Interval		Pr(>|z|)
		Lower Boundary	Upper Boundary	
**Unadjusted**				
Baseline CAC score (log CAC +1) per log increase	1.313	1.178	1.465	<0.001
**Model 1: adjusted for age**				
Baseline CAC score (log CAC +1) per log increase	1.29	1.156	1.44	<0.001
**Model 2: adjusted for Model 1 + diabetes + ASCVD + systolic blood pressure**				
Baseline CAC score (log CAC +1) per log increase	1.3343	1.1881	1.499	<0.001
**Model 3: adjusted for Model 2 + PWV + LVMI**				
Baseline CAC score (log CAC +1) per log increase	1.3577	1.2165	1.515	<0.001
**Model 4 adjusted for Model 3 + use of calcium-free phosphate binder**				
Baseline CAC score (log CAC +1) per log increase	1.1512	1.0157	1.3047	0.027

**Table 6:** Predictors of all-cause mortality among individuals with evidence of CAC progression.

Predictors of All-Cause Mortality (Cox Model)—Non-Progressor Subjects *n* = 137 (54 Fatalities)				
Model	HR	95% Confidence Interval		Pr( > |z|)
		Lower Boundary	Upper Boundary	
**Unadjusted**				
Baseline CAC score (log CAC +1) per log increase	1.058	0.9638	1.16	0.237
**Model 1: adjusted for age**				
Baseline CAC score (log CAC +1) per log increase	1.036	0.9438	1.137	0.456
**Model 2: adjusted for Model 1 + diabetes + ASCVD + systolic blood pressure**				
Baseline CAC score (log CAC +1) per log increase	1.073	0.9738	1.182	0.154
**Model 3: adjusted for Model 2 + PWV + LVMI**				
Baseline CAC score (log CAC +1) per log increase	1.069	0.9663	1.182	0.197
**Model 4 adjusted for Model 3 + use of calcium-free phosphate binder**				
Baseline CAC score (log CAC +1) per log increase	1.116	0.9993	1.246	0.051

ASCVD, atherosclerotic cardiovascular disease, defined if any of the following clinical data were reported: a history of cerebrovascular disease, peripheral vascular disease, angina pectoris, a history of myocardial infarction, aortic aneurysm or a history of percutaneous coronary angioplasty with or without stenting. PWV: pulse wave velocity; LVMI: left ventricular mass index; HR: hazard ratio.

## Data Availability

Due to data protection regulation, data regarding the independent study are not publicly available.

## Supplementary Materials

The following are available online at https://www.mdpi.com/article/10.3390/cells10051091/s1, Figure S1: Coronary Artery Calcification (CAC) stratification at study inception(A) and according to status at study completion(B), Table S1: comparisons between subjects enrolled in Independent study included and excluded from current analysis. Table S2: Independent predictors of all-cause mortality in the study cohort 1. Variables forced into the Cox model were selected based on available evidence. Table S3: The most parsimonious model (selected via a stepwise method) to predict all-cause mortality.
