# Supplementary material for: Vascular Calcification Progression Modulates the Risk Associated with Vascular Calcification Burden in Incident to Dialysis Patients
Source: Cells. 2021 May 3;10(5):1091. doi: 10.3390/cells10051091 (PMC8147653; doi:10.3390/cells10051091)
Supplement: Supplementary file 1 [file cells-10-01091-s001.zip › cells-1163499-supplementary.pdf]

Supplemental material

Supplemental Figure S1. Coronary Artery Calcification (CAC) stratification at study inception(A) and according to status at study completion(B).

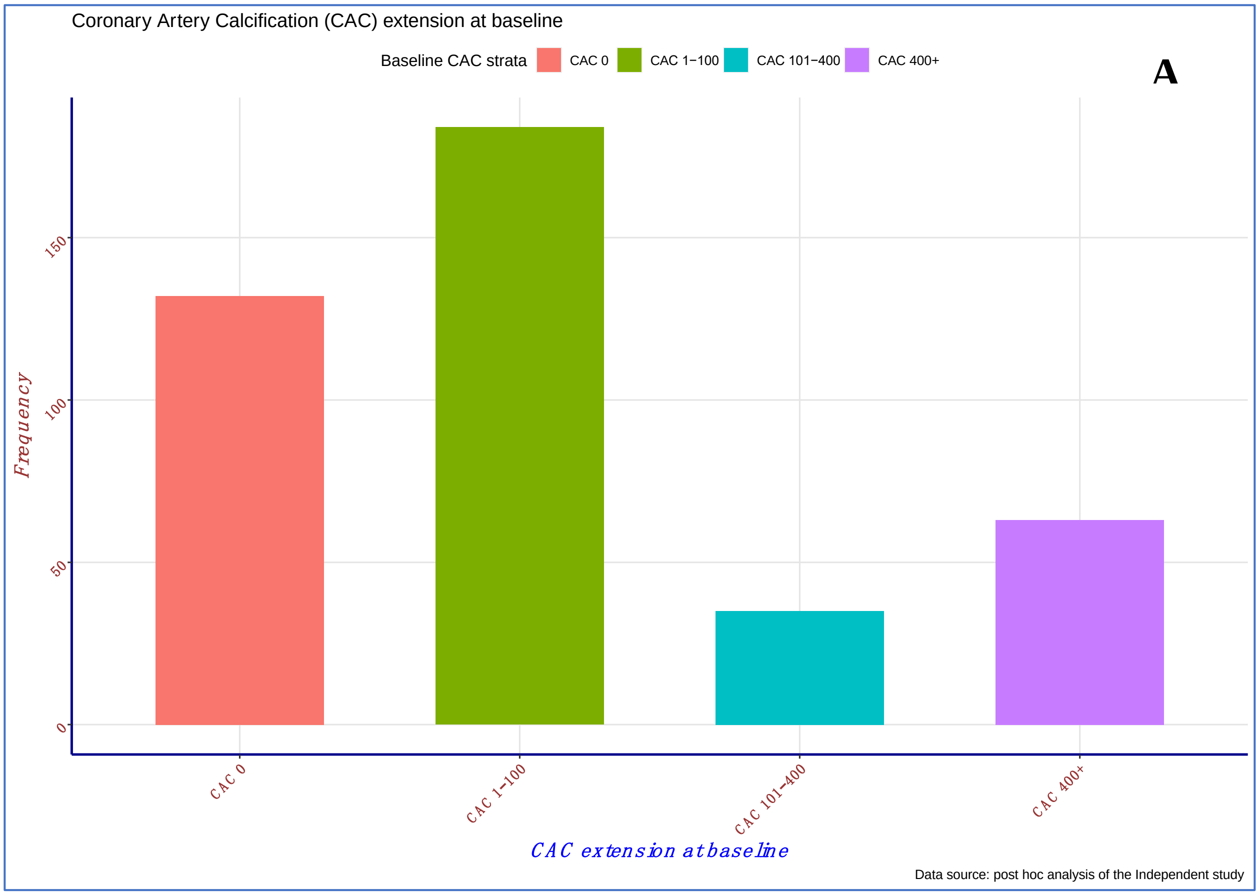

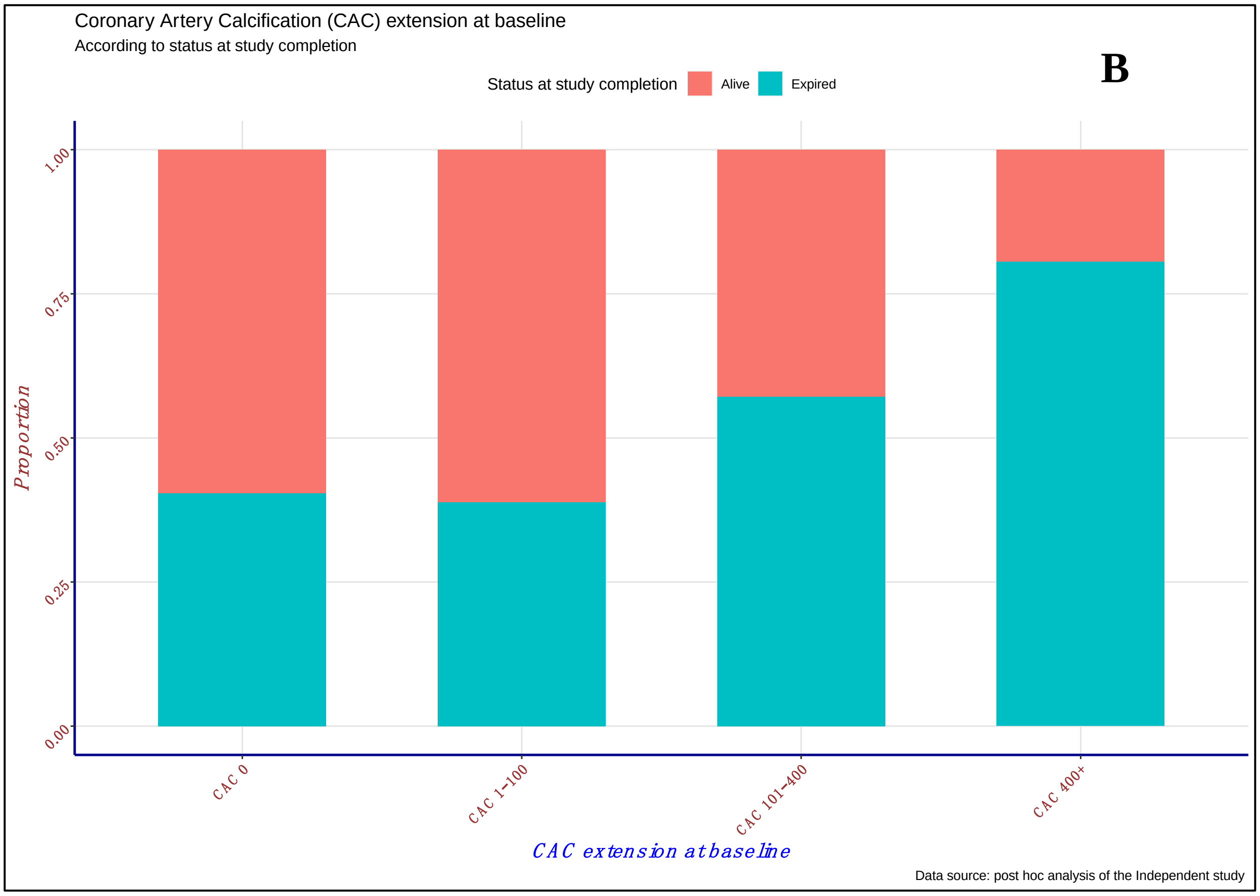

Coronary Artery Calcification (CAC) extension at baseline
According to status at study completion
B
Status at study completion
Alive
Expired
Proportion
1.00
0.75
0.50
0.25
0.00
CAC 0
CAC 1–100
CAC 101–400
CAC 400+
CAC extension at baseline
Data source: post hoc analysis of the Independent study

Supplemental Table S1: comparisons between subjects enrolled in Independent study included and excluded from current analysis

Variable	Total (n=466) Mean (SD)[n]	Included (n=414) Mean (SD)[n]	Excluded (n=52) Mean (SD)[n]	P-Value
Age (years)	65.6 (14.79)[466]	65.3 (14.83)[414]	68.3 (14.33)[52]	0.157
**Body Weight (kg)**	**69.2 (14.09)[466]**	**70.7 (13.72)[414]**	**57.5 (11.23)[52]**	**<0.0001**
Male sex	49.1% [229]	48.8% [202]	51.9% [27]	0.781
**ASCVD (yes vs no)**	**35% [163]**	**32.6% [135]**	**53.8% [28]**	**0.004**
**Diabetes**	**30.9% [144]**	**28.3% [117]**	**51.9% [27]**	**0.001**
Systolic blood pressure (mmHg)	137.1 (17.7)[466]	137 (18.01)[414]	138 (15.15)[52]	0.663
Diastolic blood pressure (mmHg)	76 (9.37)[466]	76.1 (9.13)[414]	75.2 (11.16)[52]	0.585
CAC Agatston score (unit)	256.9 (715.61)[466]	273.7 (728.84)[414]	123.4 (589.07)[52]	0.096
**CAC strata**	**n=466**	**n=414**	**n=52**	**<0.0001**
**CAC 0**	**29.4% [137]**	**31.9% [132]**	**9.6% [5]**	
**CAC 1-100**	**48.3% [225]**	**44.4% [184]**	**78.8% [41]**	
**CAC 101-400**	**8.6% [40]**	**8.5% [35]**	**9.6% [5]**	
**CAC 400+**	**13.7% [64]**	**15.2% [63]**	**1.9% [1]**	
Pulse Wave Velocity (m/sec)	8.8 (2.7)[466]	8.7 (2.49)[414]	9.3 (3.99)[52]	0.278
LVMI (g/cm2)	150.4 (46.96)[466]	149.3 (45.66)[414]	159 (56.04)[52]	0.235
QTc (msec)	407.1 (33.04)[466]	407.6 (32.73)[414]	402.8 (35.44)[52]	0.358
QTd (msec)	26.8 (11.5)[466]	26.6 (11.66)[414]	27.7 (10.13)[52]	0.486

Table legend: ASCVD: atherosclerotic cardiovascular disease defined if any of the following clinical data was reported: history of cerebrovascular disease; peripheral vascular disease; angina pectoris; history of myocardial infarction; aortic aneurysm; history of percutaneous coronary angioplasty with or without stenting; LVMI: Left ventricular mass index; CAC: coronary artery calcification

Supplemental Table S2: Independent predictors of all-cause mortality in the study cohort 1. Variables forced into the Cox model were selected based on available evidence.

**Predictors of all-casue mortality (Cox model) - All subjects n =414 (106 fatalities)**				
**Model 4 adjusted for model 3 + use of calcium free phosphate binder**				
		95% Confidence interval		
	HR	Lower Boundary	Upper Boundary	Pr(>|z|)
**Baseline CAC score (log CAC +1) per log increase**	**1.129**	**1.0114**	**1.2596**	**0.03055**
CAC progression (yes vs no)	1.9591	0.9214	4.1652	0.08058
**Age (years)**	**1.023**	**1.0059**	**1.0399**	**0.00797**
**Diabetes (yes vs no)**	**6.285**	**4.0267**	**9.8091**	**5.84E-16**
ASCVD (yes vs no)	1.2216	0.8158	1.8294	0.3312
Systolic Blood Pressure (mmHg)	1.0107	0.9992	1.0223	0.06747
**Pulse Wave Velocity (m/sec)**	**1.114**	**1.0429**	**1.1906**	**0.00136**
LVMI (g/cm2)	1.0029	0.9977	1.0081	0.27687
**Calcium-free phosphate binder vs calcium-containing phosphate binders**	**0.211**	**0.1257**	**0.3551**	**4.44E-09**
Interaction term (baseline CAC)*(CAC progression)	0.96	0.8255	1.1164	0.59595

Table legend: ASCVD atherosclerotic cardiovascular disease defined if any of the following clinical data was reported: history of cerebrovascular disease; peripheral vascular disease; angina pectoris; history of myocardial infarction; aortic aneurysm; history of percutaneous coronary angioplasty with or without stenting; LVMI: Left ventricular mass index; CAC: coronary artery calcification; HR: Hazard ratio.

Supplemental Table S3: The most parsimonious model (selected via a stepwise method) to predict all-cause mortality.

Variable	HR	lower .95	upper .95	Pr(>|z|)	
**log(CAC score) per 1 log increase**	**1.730**	**1.447**	**2.069**	**0.000**	***
**Pulse Wave velocity (m/sec)**	**1.0968**	**1.0082**	**1.193**	**0.03158**	*
Age (years	1.0167	0.9967	1.037	0.10152	
**Diabetes, yes vs no**	**3.1042**	**1.4553**	**6.622**	**0.00338**	**
ASCVD, yes vs no	0.5692	0.282	1.149	0.11584	
Systolic blood pressure (mmHg)	1.0103	0.9966	1.024	0.13968	
Use of calcium based phosphate binder, yes vs no	2.6029	0.8045	8.421	0.11029	
Use of Calcium channel blockers	1.6822	0.9516	2.974	0.07356	.

Table legend: ASCVD atherosclerotic cardiovascular disease defined if any of the following clinical data was reported: history of cerebrovascular disease; peripheral vascular disease; angina pectoris; history of myocardial infarction; aortic aneurysm; history of percutaneous coronary angioplasty with or without stenting.
